# Supplementary material for: Learning curve and surgical outcome of robotic assisted colorectal surgery with ERAS program
Source: Sci Rep. 2022 Nov 29;12:20566. doi: 10.1038/s41598-022-24665-w (PMC9709162; doi:10.1038/s41598-022-24665-w)
Supplement: Supplementary file 2 — Supplementary Information 2. [file 41598_2022_24665_MOESM2_ESM.zip › RAL Raw data-Ñ[▒K/123/OP N_ñw╝╨░O▒Kñσ.pdf]

W83 / W83

臺中榮民總醫院
手術室護理記錄

[redacted]

基本資料

- 室別： 開刀房-17室，手術科別： CRS，手術類別： 常規，日期： 2021/11/02
- 進入時間： 1225 ； 麻醉開始時間： 1245 ； Time Out時間： 1354 ； 劃刀時間： 1355 ； 手術結束時間： 1745 送出時間： 1810 ；
- 麻醉方式： GE
  診斷：s/p RRP 2007 with adhesion.
  手術名稱：Robot Assisted Colectomy(L)
  手術局部用藥：nil
  術前預防性抗生素： 無
- 手術醫師： 林俊余，陳瑢夫，林易萱，蘇偉翔
- 麻醉醫師： VS 齊光彪
-
  - 刷手護士A ： 歐真勤，時間： 1225，接替刷手一： 林彥翔，時間： 1300，接替刷手二： 陳盈妤，時間： 1500
  - 流動護士A ： 朱淨慧，時間： 1225，接替流動一： 呂昭蓉，時間： 1800
- 更多接替及流動護士： 接替刷手三： 劉雅汶，時間： 1700

皮膚狀況

A-V Shunt： 無
術前： 完整
記錄者： 上次簽章: 朱淨慧
術後： 完整
記錄者： 呂昭蓉

手術準備

手術臥式 單一手術臥式

-
  - 手術臥式： 仰臥
  - 使用附件： 溫毯 38 ℃，其它： 腳踝墊x2
  - 皮膚準備： 10%Aq Betadine，Hibiscrub，2% Chlorhexidine
  - 電燒： 使用電燒: 電極片位置： 其它： 導電軟墊，雙極電燒，記錄者： 上次簽章: 朱淨慧

止血帶T： 無

植入物

無

標本送檢

- 細菌培養 無
- 細胞學 無
- 冰凍切片 無
- 病理標本 無
- 截肢 無

標本交付 無

傷口術後敷料

傷口敷料： 傷口敷料： 無

- 請於圖上標示部位：

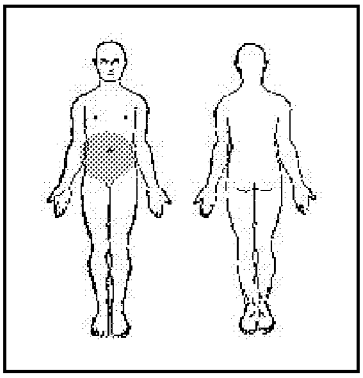

- 傷口數料： menport

導管

導管	天數	導管資訊	插入日期	插入時間	更換日期	拔除日期	拔除時間	拔除原因	預計拔除/更換	預計拔除/更換日期
Double Lumen Hemodialysis Catheter(1)	1	號碼:8 Fr.; 深度:15cm; 部位:jugular (右); 象限:RU;	2021/11/02	1250						
Sump NG Tube(1)	1	號碼:16 Fr.; 深度:58cm; 部位:胃;	2021/11/02	1255						
A-line(1)	1	號碼:20 G; 部位:右手;	2021/11/02	1300						
Foley(1)	1	號碼:16 Fr.; 部位:膀胱;	2021/11/02	1305						

用物計數

- 手術中出血量： minimal c.c.
- 手術中尿量： 1550 c.c.
-
    - 其它： 無

用物計數： 多切口同時縫合

- 1.器械： 正確，2.縫針： 正確，3.刀片： 正確，4.紗布： 正確，5.腹部墊： 未使用，6.紗球： 未使用，7.PEANUTS ： 未使用，8.紗條： 未使用，9.絲帶： 未使用，10.針頭： 未使用，11.Vessel loop： 未使用，12.Boots： 未使用，13.Jelco： 未使用，14.棉條： 未使用，
    - 第一次：
        - 刷手護士 ： 劉雅汶
        - 流動護士： 上次簽章: 朱淨慧
        - 時間： 1730

,

- 第二次：
    - 刷手護士 ： 劉雅汶
    - 流動護士： 上次簽章: 朱淨慧
    - 時間： 1735

,

- 第三次：
    - 刷手護士 ： 劉雅汶
    - 流動護士： 上次簽章: 朱淨慧
    - 時間： 1740

備註

備註： Davinci XI 0度 scope No.1
Davinci XI 30度 scope No.2
達文西上機時間:14:50
達文西下機時間:17:30

- 記錄者： 上次簽章: 朱淨慧
- 時間： 1425

填寫護理人員 呂昭蓉

正式摘要

W83 / W83

doctorRank:B
